# The Population Decline of *Gyps* Vultures in India and Nepal Has Slowed since Veterinary Use of Diclofenac was Banned

**DOI:** 10.1371/journal.pone.0049118

**Published:** 2012-11-07

**Authors:** Vibhu Prakash, Mohan Chandra Bishwakarma, Anand Chaudhary, Richard Cuthbert, Ruchi Dave, Mandar Kulkarni, Sashi Kumar, Khadananda Paudel, Sachin Ranade, Rohan Shringarpure, Rhys E. Green

**Affiliations:** 1 Bombay Natural History Society, Mumbai, India; 2 Bird Conservation Nepal, Kathmandu, Nepal; 3 Royal Society for the Protection of Birds, Sandy, Bedfordshire, United Kingdom; 4 Conservation Science Group, Department of Zoology, University of Cambridge, Cambridge, United Kingdom; University of Bern, Switzerland

## Abstract

Populations of oriental white-backed vulture (*Gyps bengalensis*), long-billed vulture (*Gyps indicus*) and slender-billed vulture (*Gyps tenuirostris*) crashed during the mid-1990s throughout the Indian subcontinent. Surveys in India, initially conducted in 1991–1993 and repeated in 2000, 2002, 2003 and 2007, revealed that the population of *Gyps bengalensis* had fallen by 2007 to 0.1% of its numbers in the early 1990s, with the population of *Gyps indicus* and *G. tenuirostris* combined having fallen to 3.2% of its earlier level. A survey of *G. bengalensis* in western Nepal indicated that the size of the population in 2009 was 25% of that in 2002. In this paper, repeat surveys conducted in 2011 were analysed to estimate recent population trends. Populations of all three species of vulture remained at a low level, but the decline had slowed and may even have reversed for *G. bengalensis*, both in India and Nepal. However, estimates of the most recent population trends are imprecise, so it is possible that declines may be continuing, though at a significantly slower rate. The degree to which the decline of *G. bengalensis* in India has slowed is consistent with the expected effects on population trend of a measured change in the level of contamination of ungulate carcasses with the drug diclofenac, which is toxic to vultures, following a ban on its veterinary use in 2006. The most recent available information indicates that the elimination of diclofenac from the vultures’ food supply is incomplete, so further efforts are required to fully implement the ban.

## Introduction

Vultures of the genus *Gyps* are obligate scavengers on the carcasses of dead vertebrates; most commonly wild and domesticated ungulates. Throughout the Indian subcontinent, populations of oriental white-backed vulture (*Gyps bengalensis*), long-billed vulture (*Gyps indicus*) and slender-billed vulture (*Gyps tenuirostris*) declined rapidly, beginning in the 1990s [1]–[5]. Previous road transect surveys in India, initially conducted in 1991–1993 and repeated in 2000, 2002, 2003 and 2007, revealed that, by 2007, *G. bengalensis* had fallen to 0.1% of its numbers in the early 1990s, with populations of *G. indicus* and *G. tenuirostris* combined having fallen to 3.2% of their earlier level [5]. Marked declines of *G. bengalensis* and *G. indicus* have been reported from Pakistan [1] and of *G. bengalensis* and *G. tenuirostris* from lowland regions of Nepal [6], [7].

Populations of several species of vultures and other large scavenging birds worldwide have declined because of reductions of food availability [8], collisions with man-made structures [8], contamination with remains of spent lead ammunition [9] and poisoning of vultures which may be deliberate or a side-effect of poisoning mammalian predators and scavengers [10], [11]. However, the declines of Asian Gyps vultures have been unusually rapid and research has indicated the involvement of a novel cause. Veterinary use of the non-steroidal anti-inflammatory drug (NSAID) diclofenac is the main, and perhaps the only, cause of these population declines. Evidence concerning the relative importance of this and other potential causes has been presented in detail elsewhere [3], [12], [13]. Vultures are exposed to diclofenac when they feed from carcasses of livestock that have died within a few days of treatment and still contain residues of the drug [12]. Vultures that consume sufficient tissue from such carcasses die from the effects of diclofenac-induced kidney failure. Green et al. [3] estimated that less than 0.8% of ungulate carcasses available to foraging vultures would need to contain a lethal dose of diclofenac for this to have caused the observed population declines. They also found that the high proportion of *G. bengalensis* and *G. indicus* found dead in the wild which had severe visceral gout was consistent with diclofenac poisoning being the main or sole cause of the population declines. In 2004–2005, the proportion of carcasses of domesticated ungulates in India contaminated with diclofenac and the concentration of the drug in their tissues were sufficient to have caused vulture declines at the observed rates without the involvement of any other factor [14]. Efforts to achieve the voluntary withdrawal of use of veterinary diclofenac began in 2004. The licence to manufacture veterinary formulations of diclofenac was withdrawn by the Drug Controller General of India via a letter dated 11 May 2006 addressed to all the state drug controllers. Similar bans on the veterinary use of diclofenac were also introduced in Nepal and Pakistan in 2006 and in Bangladesh in 2010. The toxicity of diclofenac to vultures and the evidence of its effect on their populations were the reasons for withdrawal. Vultures are important to human well-being in the Indian subcontinent, because they dispose of the carcasses of ungulates which would otherwise be left to rot or to provide food for the growing population of feral dogs, which cause health risks and nuisance.

Previous studies have reported successive stages of the vulture population declines in the Indian subcontinent, with the most recent published analysis of widespread population surveys in India, which contains most of the vulture population of the subcontinent, having dealt with all of the road transect surveys in that country up to 2007 [5]. Had the rates of decline reported then continued, the most rapidly declining species, *Gyps bengalensis*, would by now be reduced to very low numbers. To update the previous analyses of population survey results, we report here the results of vulture surveys across a large area of northern India and Nepal during 2011, which are analysed in combination with previous surveys which followed the same methods and transects. We use these results to estimate the recent changes in the population trends of the three critically endangered species of *Gyps* vultures and compare these changes with those expected from changes in the prevalence and concentration of diclofenac in ungulate carcasses since the ban on its veterinary use was introduced.

## Methods

### Vulture Surveys

In India, vultures were counted on road transects widely distributed across northern, central, western and north-eastern India in 2011. Transects were positioned in and near National Parks and other nature reserves (99 transects, total length 5 221 km) and also along roads distant from such protected areas (55 transects, total length 10 296 km). Many of them were transects used in a previous survey carried out in the three-year period 1991–1993, which will be referred to as the 1992 survey for brevity. The survey was repeated in 2000, 2002, 2003 and 2007, with additional transects being added in some years. Routes followed in 2011 were the same as in previous surveys. Each transect was driven in a motor vehicle by a driver and observer. Transects were driven between 08∶00 and 17∶00 local time at 10–20 km h^−1^ in protected and surrounding areas, and 50 km h^−1^ on highways., with the speed being kept similar in different surveys of the same transect. The transects were conducted in the period March - June in all six surveys, except that surveys were also conducted in a few areas in December - January in the 1991–1993 survey. However, results from that survey are not used in the analyses of changes in the rate of population decline presented in this paper. For that, we only use data from 2000 onwards, for which the survey period and timing are comparable in all surveys. Vultures seen by the observer within 500 m on either side of the route were recorded. Vultures were identified to species, but *G. indicus* and *G. tenuirostris* were only distinguished in the 2002, 2003, 2007 and 2011 surveys because they were only recognized as separate species in 2001 [15]. In the 1992 survey, only vultures in groups of five or more were counted because they were very numerous then, but in 2000, 2002, 2003, 2007 and 2011 all vultures seen were recorded. The numbers of transects surveyed in each of the years 1992, 2000, 2002, 2003, 2007 and 2011 were 92, 98, 159, 149, 165 and 154 respectively. The total length of transects driven in 2011 was 15 518 km. Further details of the methods are given by Prakash et al. [4] and there is a map showing transect locations in ref [14].

In Nepal, road transects were undertaken in the western half of Nepal (Far Western, Mid Western and Western development regions) in May in each of seven years between 2002 and 2011. The transect route was the same in each year and covered 638 km, following the main East – West Highway traversing the lowland districts of Nawalparasi, Rupandehi, Kapilbastu, Dang, Banke, Bardia, Kailali and Kapilbastu, where resident *Gyps* vultures were formerly most abundant [6]. A vehicle was driven at 20 km h^−1^ and all flying and perching vultures seen within 1 km of the road were recorded and identified to species. Two observers facing opposite directions carried out the survey between 07∶00 and 17∶00 local time. The driver did not count vultures.

Although the plumage of some of the individual vultures observed was seen well enough to allow them to be allocated to broad age categories, we consider that the consistent recording of plumages was possible for an insufficient number of birds to warrant any analysis of changes in the age compostion of vultures.

No specific permits were required for the field studies described. All transects in Nepal and most in India were conducted on roads and tracks accessible to the public. In India, some transects were conducted within protected areas. Access permission was obtained in these cases. Vultures were observed and counted from within a vehicle on a road or track. Hence, even though the vultures are protected species, the counts conducted did not entail any disturbance of them which requires specific permission.

### Calculation of Estimates of Change in Population

Not all of the transects in India were surveyed in all years. Some were surveyed for the first time only after several previous surveys had been carried out elsewhere, while others ceased to be surveyed after a few years of coverage or had gaps in coverage. To allow for this variation in survey coverage, we fitted log-linear Poisson regression models that allow for the effects of changing composition of the sample of transects.

In the Poisson regression analyses, the vulture count on each transect was treated as the dependent variable. The identity of the transect and the survey year were modelled as factors affecting the count. Including the effect of the transect in this way allows for changes across years in the representation of transects in the surveyed sample. Models were fitted in GLIM 4, with a Poisson error term and a logarithmic link function. The regression coefficients *k_i_* representing the year effects are the logarithms of the abundance of birds in survey year *i* expressed as a proportion of the abundance during the first survey year. Hence, exp(*k_i_*) gives an index of population density in the *i*th year, which is the population in that year relative to that in the first year of the series. We obtained 95% confidence intervals for the population index values using a bootstrap method. We took random samples of *m* transects, with replacement, from the data from the *m* transects surveyed more than once during a particular time period. We then fitted the log-linear Poisson regression model for this bootstrap sample and recorded the value of exp(*k_i_*) for each of the survey years. This procedure was repeated 1 000 times and the central 950 of the bootstrap estimates were used to define the 95% confidence interval of each of the population index values. In some analyses, we included all the surveys (1992–2011), but in others only those during the periods 2000–2011 or 2002–2011. In particular, we were only able to analyse data for *G. indicus* and *G. tenuirostris* separately for 2002–2011, the period when these two species were separately recorded. Over the periods beginning in 1992 and 2000, we modelled the population index for the combined counts of these two species. However, because the total numbers of *G. tenuirostris* were much smaller than those of *G. indicus* in all surveys since 2002 (<2%), the index for the two species together can be regarded as approximately representing the situation for *G. indicus* alone.

To test whether the annual rate of population change had altered over time, we fitted Poisson regression models with a logarithmic link function and transect as a factor, as before, but with the effect of year modelled as a continuous explanatory variable *t*; the number of years elapsed since the first survey of the series being used. We did this only for the period 2000–2011; because we considered it unwise to estimate the average annual rate of population decline over the period 1992–2011, given that the rapid vulture population decline began at an uncertain time between 1992 and 2000. We wished to test whether the rate of population change remained constant or changed within the period 2000–2011 and therefore fitted Poisson regressions with the independent variable *t*, and compared the results with the quadratic regression with both *t* and *t* ^2^, and the cubic regression with *t*, *t* ^2^ and *t* ^3^. If the inclusion of the higher order polynomial terms significantly improved the fit of the regression, this would indicate that the rate of population change varied over time. We tested this possibility using *F* tests. Likelihood-ratio tests were not performed because vultures often occur in groups, leading to counts being overdispersed. We also estimated the average annual rate of population decline, as a percentage, in the period between two consecutive surveys at times *t_i_* and *t_j_*, as 100(1-exp((*k_j_*-*k_i_*)/(*t_j_*-*t_i_*)) and obtained 95% confidence limits of these rates by the bootstrap method described above.

In Nepal, the same transect route was followed in all seven surveys so the regression model did not need to allow for changes in coverage. Only *G. bengalensis* was recorded in sufficient numbers for analysis. We fitted Poisson regressions with the time elapsed since the first survey in 2002 as a continuous explanatory variable *t*, and compared the results of this with the quadratic regression with both *t* and *t* ^2^, and the cubic regression with *t*, *t* ^2^ and *t* ^3^ by means of *F* tests, as described above.

### Calculation of Changes in Demographic Rates Required to Cause the Observed Changes in the Rate of Population Decline

Our road transect survey results, described below, indicated that the rate of the population decline of both vulture species had slowed since the rapid declines reported previously for the period 2000–2003 [3]. We wished to investigate the magnitude of the changes in demographic rates which would be needed to account for the observed changes in population size. We do not know of any reliable time series of direct measurements of demographic rates of vultures in the Indian subcontinent, so we can only do this by this using simulation modelling. We adapted a previously published simulation model of Asian vulture populations [3] in which the annual survival rates of adults and pre-adults and the average number of fledglings produced per adult pair were assumed to take values which would keep the population stable under normal conditions, but which allowed an additional cause of mortality to affect the daily survival probability of all full-grown birds (adults and pre-adults) to the same extent. The production of fledglings was also assumed to be affected by this additional mortality because of deaths of breeding adults: it being assumed that the death of either member of a breeding pair would cause breeding failure. This model was originally developed to examine the potential effects of diclofenac poisoning, but its results are relevent to any novel cause of mortality of full-grown birds. Full details of the model are given elsewhere [3].

We adapted the model in two ways. The first modification was to convert it to simulate the total population size of all full-grown birds in annual steps for each of the years 2000 to 2012. Adult survival rate has not been measured in Asian *Gyps* vultures, but we followed the previous study [3] in assuming, based upon studies of similar species, that a plausible range of the value of this parameter *S_0_* for these species, before the recent population decline began was 0.90 to 0.97. We performed simulations for both of these values. Survival from fledging to the age of first breeding (assumed to be 5 years, as in [3]) was assumed to result from successive equal annual pre-adult survival probabilities in each year of life. Survival over the whole period from fledging to first breeding was found to be 0.727 in a study of *Gyps fulvus*, compared with annual adult survival of 0.975 [16], and we took the ratio of these two rates (0.745) to generate values of survival from fledging to first breeding from the two assumed adult survival rates given above.

The second modification of the population model was to change the assumed number of fledglings produced per adult pair in the absence of the addition source of mortality so as to allow the population to grow in the absence of that mortality. In the original version of the model, the population was assumed to be stable in the absence of the additional mortality [3]. An assumption that the vulture population should be able to grow if the additional mortality is removed is realistic given that the populations of all *Gyps* species in the Indian subcontinent have fallen to very low levels compared with those in the early 1990s and marked density-dependent reduction of intra-specific competition for food and other resources would be expected to have occurred. However, it is necessary to make an estimate of how large the population growth rate at low density might be if the additional source of mortality was removed. We did this by assuming that it was the maximum annual rate of population growth λ_max_ and used the demographic invariant method of Niel and Lebreton [17] which allows the calculation of λ_max_ from the annual adult survival rate and age of first breeding *B* using their equation 17. Having obtained values for λ_max_,We then used a Leslie matrix model and the assumptions about the annual survival of adults and pre-adults given above to calculate the number of fledglings produced per adult pair required to give these values of λ_max_, with the rate of additional mortality set to zero.

We used the simulation model and the values of the annual adult and pre-adult survival and breeding success in the absence of additional mortality generated in the steps described above and added daily additional mortality due to a new cause. We used the model to obtain the daily rate of additional mortality of full-grown birds which would cause the simulated population to decline during the period 2000–2003 at the rates previously reported for India during that period (λ = 0.520 for *Gyps bengalensis* and λ = 0.775 for *G. indicus* [3]). We applied this rate of additional mortality up to 2004. From 2005 onwards, we assumed that the reduced level of adult survival caused by the additional mortality recovered linearly in equal annual steps over a recovery period of duration *T* years, until it reached the value of adult survival assumed when there is no additional mortality. We chose 2005 onwards for the beginning of the change because this was when actions began to be taken to reduce the exposure of wild vultures to diclofenac. The rates of additional daily mortality needed to cause these simulated changes in adult survival were calculated and this was used to obtain year-specific values of the annual survival of pre-adults and breeding success during the period of change. We then varied *T* iteratively until the ratio of the simulated total vulture population in 2011 to that in 2007 matched the observed ratio of estimates of abundance in the two road transect surveys in those two years. This procedure gave the simulated changes in survival rates and breeding success that would result in the observed population changes between 2007 and 2011.

### Calculation of the Change in the Rate of Population Decline Expected from the Change in Diclofenac Contamination Since the Ban on its Veterinary Use in India

We used the used a simulation model of the impact of diclofenac contamination on the population trend of vultures described by Cuthbert et al. [18] to determine the expected effect of changes in contamination on the rate of change of the population of *G. bengalensis* in India. There is no equivalent model for *G. indicus* or *G. tenuirostris* because there are no experimental determinations of the toxicity of diclofenac to these species. Cuthbert and colleagues estimated the expected rate of vulture population decline from two surveys of diclofenac in ungulate carcasses conducted just after the ban (T2– samples collected April – December 2006, T3– January 2007– December 2008) and expressed these rates relative to the expected rate of decline they estimated from ungulate liver samples collected before the ban (T1– May 2004– July 2005). They found that the ratio of the expected rate of decline at the time of survey T2 to that at survey T1 was 0.903–0.924, with the range of values being based upon four sets of assumptions about the interval between vulture meals and adult survival. The equivalent ratio for survey T3 relative to T1 was 0.351–0.413 (see Table 5 of ref. [18] for details and confidence limits). Hence, the population of *G. bengalensis* was still expected to be declining at the time of the most recent diclofenac survey (T3–2007–2008), but the expected annual rate of decline had fallen by more than half compared with the expected decline rate before the ban. To compare the results of our road transect surveys in India with these expectations, we used the cubic regression model of *G. bengalensis* population in relation to time described previously to estimate decline rates at the midpoints of each of the diclofenac surveys, T1, T2 and T3. We did this by calculating from the regression model the rate of decline at the average liver sampling date for each of the three surveys (T1–23 December 2004, T2–22 August 2006, T3–23 January 2008). We then took the ratio of the decline rate for T2 relative to that for T1 (T2:T1) and the equivalent ratio for T3:T1. These ratios were compared with the ratios of expected decline rates determined from diclofenac prevalence and concentration. Repeated surveys of diclofenac contamination of ungulate carcasses have not been carried out in Nepal, so a similar analysis cannot be performed for that country.

## Results

### Long-term Trend of Vulture Populations in India

**Figure 1:**
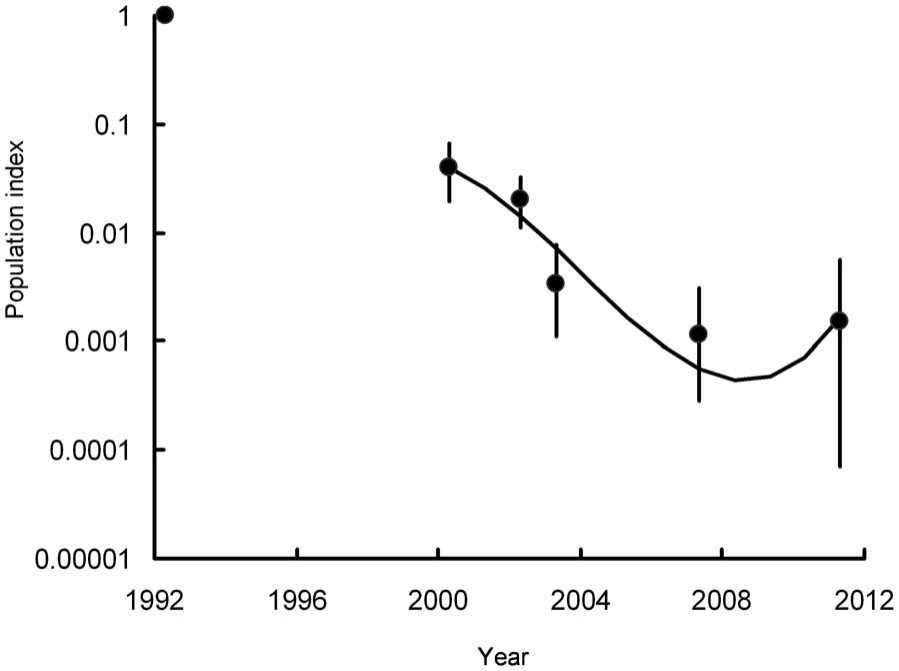
Population indices and trend of *Gyps bengalensis.* Circles show indices of population density, relative to that in 1992, estimated by log-linear Poisson regression preformed on data from six road transect surveys in India. Vertical lines show 95% bootstrap confidence limits. The curve shows the cubic log-linear population trend fitted to data for the period 2000–2011.

**Figure 2:**
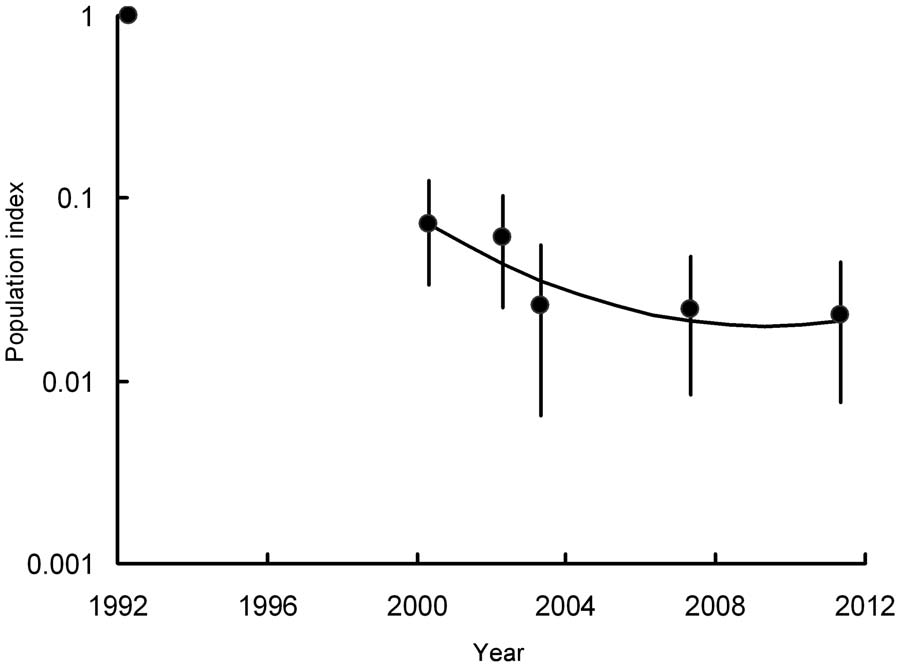
Population indices and trend of *Gyps indicus* and *G. tenuirostris* combined. Circles show indices of population density, relative to that in 1992, estimated by log-linear Poisson regression preformed on data from six road transect surveys in India. Vertical lines show 95% bootstrap confidence limits. The curve shows the quadratic log-linear population trend fitted to data for the period 2000–2011.

The log-linear Poisson regression modelling indicated highly significant population changes in all three vulture species in India over all of the time periods studied (Table 1, Figure 1, Figure 2). The population index for *G. bengalensis* in 2011 was 0.15% of that in 1992 and the index for *G. indicus* and *tenuirostris* combined was 2.29% of the 1992 value (Table 1). Even over the shorter period 2000–2011, the declines have been large, though the change for *G. indicus* and *tenuirostris* was much less than for *G. bengalensis*. The index for *G. indicus* and *tenuirostris* in 2011 was 31.4% of the 2000 value, whereas that for *G. bengalensis* in 2011 was 3.9% of the 2000 value. In 2011, the population index for *G. tenuirostris* was 48.8% of its value in 2002, when the species was first surveyed separately. However, the index for this species is based upon a small number of transects and the index value has therefore fluctuated considerably across surveys. On transects surveyed in both 2007 and 2011, totals of 80 and 99 *G. bengalensis* were counted in the two years respectively. The equivalent totals for *G. indicus* were 337 and 299 and for *G. tenuirostris*, 7 and 15.

**Table 1:** Population indices and trends of vultures.

Species	*G. bengalensis*	*G. indicus & tenuirostris*	*G. bengalensis*	*G. indicus & tenuirostris*	*G. indicus*	*G. tenuirostris*
Time period	1992–2011	1992–2011	2000–2011	2000–2011	2002–2011	2002–2011
Informative transects	121	106	81	72	38	13
Year	Population relative to that in first year of series					
2000	0.0389	0.0728				
	(0.0197–0.0669)	(0.0333–0.1241)				
2002	0.0199	0.0612	0.5147	0.8371		
	(0.0110–0.0321)	(0.0253–0.1036)	(0.2747–0.9712)	(0.3379–1.9705)		
2003	0.0034	0.0256	0.0872	0.3492	0.4207	0.1352
	(0.011–0.0079)	(0.0064–0.0552)	(0.0265–0.2231)	(0.0740–1.0712)	(0.1451–0.7595)	(0.0001–0.6190)
2007	0.0012	0.0246	0.0300	0.3369	0.4083	0.2276
	(0.0003–0.0030)	(0.0083–0.0485)	(0.0059–0.0847)	(0.1271–0.7477)	(0.1917–0.8208)	(0.0001–1.0435)
2011	0.0015	0.0229	0.0396	0.3129	0.3628	0.4877
	(0.0001–0.0057)	(0.0076–0.0446)	(0.0021–0.1294)	(0.1117–0.6107)	(0.1160–1.0725)	(0.0962–2.0000)
	Significance of variation in population index among survey years					
*F*	1114.71***	262.61***	64.96***	27.74***	6.25**	2.94*
d.f.	5,485	5,440	4,263	4,246	3,103	3,33

Indices and trends were estimated by log-linear Poisson regression from road transect counts in India. Each column shows results for a particular species and time period. Informative transects are those that were surveyed more than once and on which at least one vulture of the species concerned was recorded during the time period. Population indices are estimates of the population density as a proportion of that in the first year of the period shown at the head of each column. A 95% bootstrap confidence interval is shown for each index (in brackets). *F* tests of significance are shown with *P* values indicated as; ***P<0.001, **P<0.01, *P<0.05.

### Long-term Trend of Vulture Populations in Western Nepal

**Figure 3:**
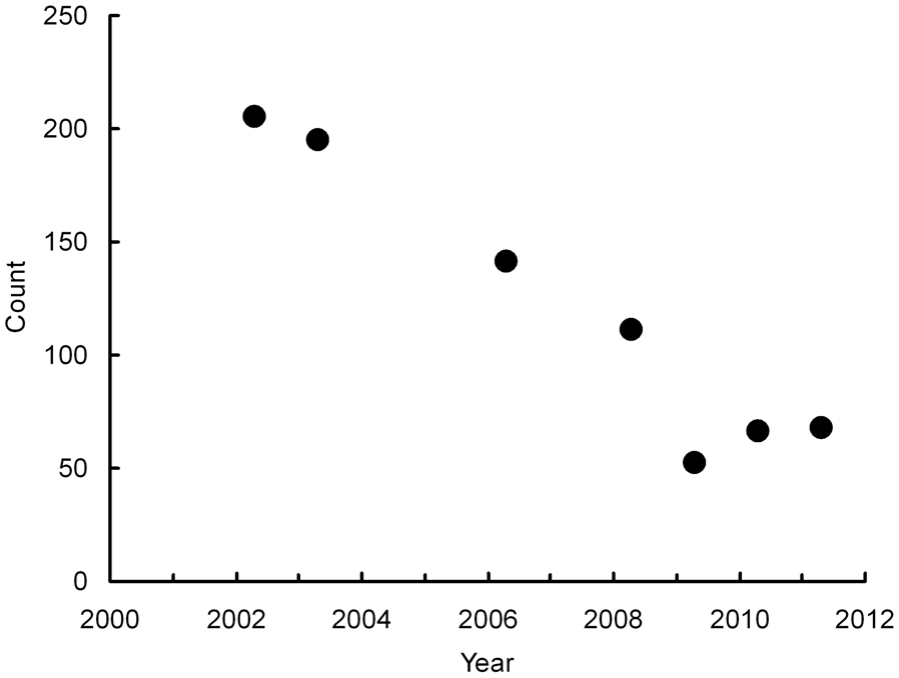
Counts of *Gyps bengalensis* on a road transect through western Nepal.

Numbers of *G. bengalensis* counted on the transect through western Nepal fell from 205 in 2002 to 52 in 2009, with declines in each of the four successive repeat surveys. Hence, the count in 2009 was 25.4% of that in 2002. The mean annual rate of population decline in this period, estimated by log-linear Poisson regression, was 13.5% per year. In 2010 and 2011 the numbers of vultures recorded increased, reaching 68 in 2011 (Figure 3), which is 33.2% of the count for 2002.

### Change in the Rate of Population Decline

**Figure 4:**
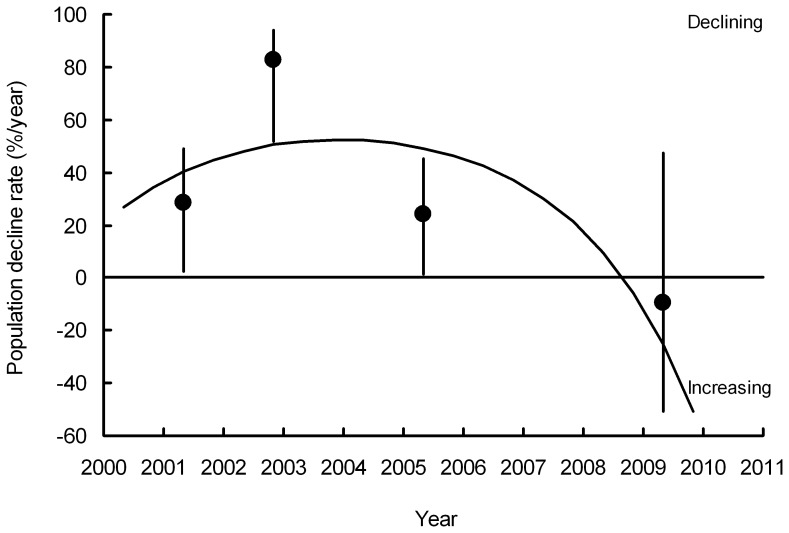
The rate of population decline (% per year) of *Gyps bengalensis* in India. Circles show average annual rates between each pair of consecutive surveys with their 95% bootstrap confidence limits (vertical lines) plotted midway between the dates of the two surveys. The curve shows the first derivative of the cubic log-linear population trend fitted to the survey data for the period 2000–2011.

**Figure 5:**
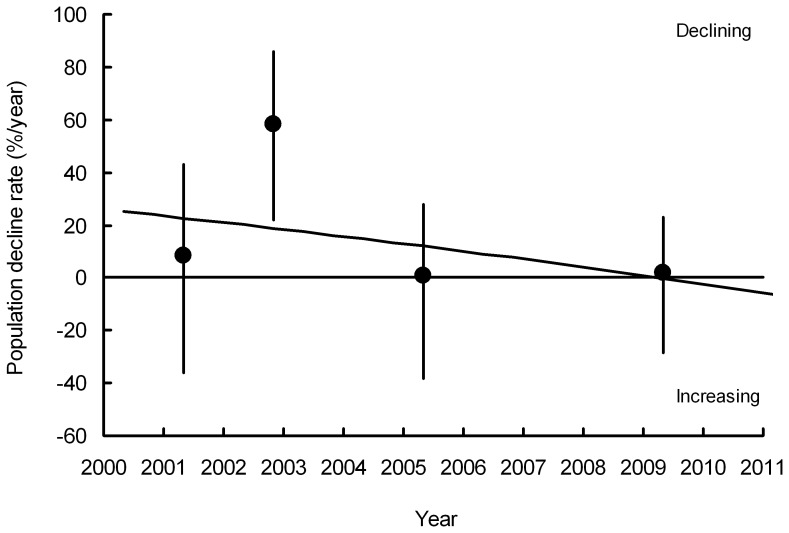
The rate of population decline (% per year) of *Gyps indicus* and *G. tenuirostris* combined in India. Circles show average annual rates between each pair of consecutive surveys with their 95% bootstrap confidence limits (vertical lines) plotted midway between the dates of the two surveys. The line shows the first derivative of the quadratic log-linear population trend fitted to the survey data for the period 2000–2011.

Inspection of graphs of rate of population change in India against year for the period 2000–2011 suggests that the annual rate of population decline of *G. bengalensis* (Figure 4) and of *G. indicus* and *tenuirostris* combined (Figure 5) have both slowed towards the end of the period. *F* tests on the polynomial functions fitted to the transect data indicated a statistically significant change in the rate of decline. For both *G. bengalensis* and for *G. indicus* and *tenuirostris* combined, a quadratic regression significantly improved the fit compared with that of the linear regression with time elapsed since the 2000 survey alone (*F_1,265_* = 14.41, *P*<0.001, *F_1,248_* = 5.21, *P* = 0.023 respectively). The quadratic regression of population index models the rate of population decline as changing linearly with time. For *G. bengalensis* the cubic regression gave a significantly improved fit over the quadratic, indicating a more complex relationship for this species between rate of population decline and time (*F_1,264_* = 8.08, *P* = 0.005). However, this was not the case for *G. indicus* and *tenuirostris* combined, for which the cubic regression did not improve the fit significantly (*F_1,247_* = 0.04, *P* = 0.834). Hence, we used the cubic regression to describe changes in population of *G. bengalensis* and the quadratic regression for *G. indicus* and *tenuirostris* (Figs 1, 2, 4, 5). For both *G. bengalensis* and for *G. indicus* and *tenuirostris* combined, these analyses indicate that the previously rapid population declines slowed in the late 2000s. The population of *G. bengalensis* may recently have begun a slow increase, though the recent estimate of trend is not significantly different from zero (Figure 4).

In western Nepal, although counts increased in 2010 and 2011 after being at their lowest level in 2009, inclusion of quadratic and cubic terms in the regression did not significantly improve model fit (*P*>0.50), so the observed cessation of the decline does not represent a statistically significant change in the rate of population change. Nonetheless, inspection of the survey data indicates a similar pattern to India, where the rate of decline has slowed significantly.

### Have Rates of Population Decline in India Slowed because Vultures now Largely Remain at Sites Whose Population has been Consistently Stable?

**Figure 6:**
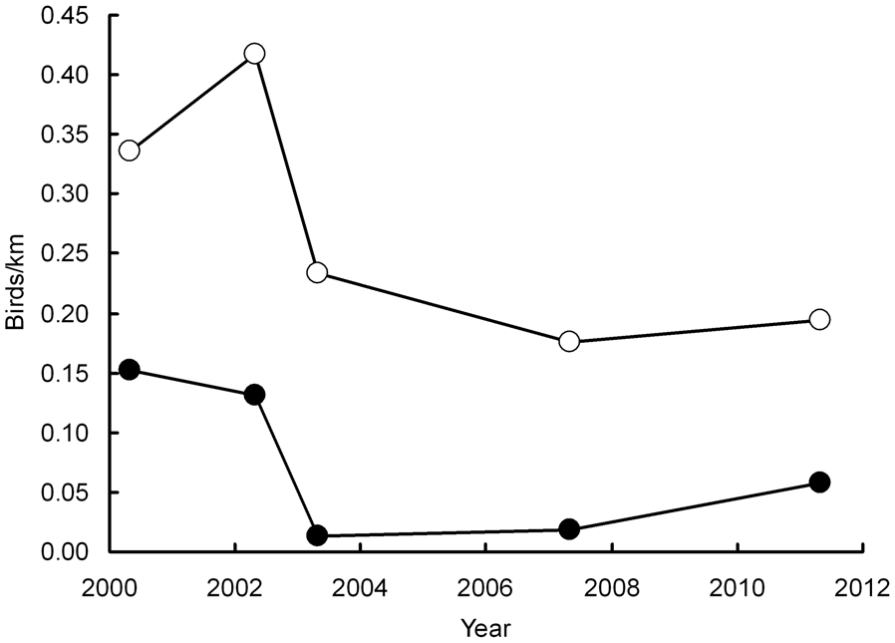
Average numbers of *Gyps bengalensis* (filled circles) and *Gyps indicus* (open circles) seen per kilometre of road transect at selected sites. Results are for transects in and near National Parks in India which contained the majority of records of these species in 2011.

The recent slowing in the apparent rate of decline of vulture populations described above might hypothetically have occurred because areas differ in the adverse circumstances that are causing the decline, and because these differences have remained constant over time with little movement of birds between them. If this was the case, vultures would progressively die out in areas with rapid declines and largely remain where the population has been stable or declining more slowly since our surveys began. Under this hypothesis, the rate of decline of the whole population would slow down even if the strength of the external cause of the decline remained the same. To test this idea using the data for India, we took road transect data collected within or adjacent to selected National Parks (NP) from which the majority of our 2011 records of *G. bengalensis* and *G. indicus* came. For *G. bengalensis* these were Corbett NP in Uttarakhand, and Pench NP, Bhandavgarh NP and Kanha NP, which are all in Madhya Pradesh. Ninety-nine percent of the *G. bengalensis* recorded in 2011 were seen at these sites. For *G. indicus* the parks were Bhandavgarh, Gir NP in Gujarat and Ranthambore NP and Desert NP, which are both in Rajasthan. Eighty-seven percent of the *G. indicus* recorded in 2011 were seen at these sites. We then combined results from all available transects for these sites, separately for each species, and calculated the number of vultures recorded per kilometre of transect for each of the surveys in 2000, 2002, 2003, 2007 and 2011. There was a change from decline to stability or population increase for both species at these subsets of sites (Figure 6). This result is consistent with there having been a reduction over time in the external cause of the decline and not with the hypothesis of differential extinction of subpopulations, each of which has a different but unchanging decline rate. However, we note that the total decline in density between 2000 and 2007 of both species in the protected areas where most of the remaining birds were recorded (88% for *Gyps bengalensis* and 48% for *G. indicus*) was smaller than the decline in the index of the whole population over this period (97% for *Gyps bengalensis* and 66% for *G. indicus*).

### Changes in Demographic Rates Required to Cause the Observed Changes in the Rate of Population Decline

Application of the demographic invariant method of Niel and Lebreton [17] resulted in estimates of the maximum annual rate of population growth of λ_max_ = 1.115 when *S_0_* was assumed to be 0.90 and λ_max_ = 1.071 when *S_0_* = 0.97. These values are similar to that determined by Niel and Lebreton from direct measurements of demographic rates for a growing population of *Gyps fulvus* [16], [17] (λ_max_ = 1.09) in Europe. The mean number of fledglings produced per pair required to achieve these values of λ_max_ were 0.991 for *S_0_* = 0.90 and 0.366 for *S_0_* = 0.97.

**Figure 7:**
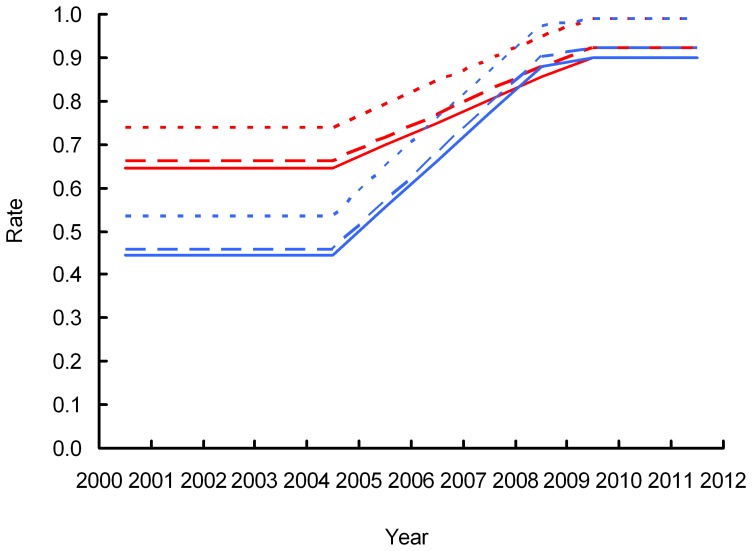
Simulated changes in the annual survival rate of adults (solid lines), the annual survival of pre-adults (dashed lines) and the mean number of fledglings per adult pair per year (dotted lines) required to account for the observed change in the population index derived from road transects between 2007 and 2011. Results are shown separately for *Gyps bengalensis* (blue) and *Gyps indicus* (red). In these simulations the annual survival of adults in the absence of additional mortality was assumed to be 0.90.

**Figure 8:**
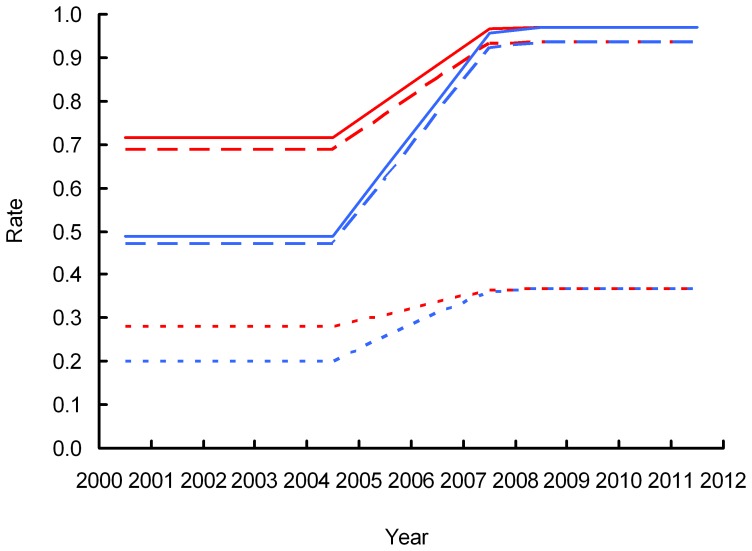
Simulated changes in the annual survival rate of adults (solid lines), the annual survival of pre-adults (dashed lines) and the mean number of fledglings per adult pair per year (dotted lines) required to account for the observed change in the population index derived from road transects between 2007 and 2011. Results are shown separately for *Gyps bengalensis* (blue) and *Gyps indicus* (red). In these simulations the annual survival of adults in the absence of additional mortality was assumed to be 0.97.

Our simulation model of the vulture population in India indicated that the duration *T* of recovery period, starting in 2005, during which annual survival increased linearly until reaching the values assumed in the absence of additional mortality was 4.2 years for *Gyps bengalensis* and 4.8 years for *G. indicus* if adult survival in the absence of additional mortality *S_0_* was assumed to be 0.90 (Figure 7), and 3.1 years for both species if *S_0_* = 0.97 (Figure 8). The simulations indicated that, to account for observed rates of population change, annual survival rates and breeding success would need to have been much more severely reduced for *Gyps bengalensis* during the period 2000–2003 when populations were declining rapidly than they were for *G. indicus*.

### Change in the Rate of Population Decline Expected from the Change in Diclofenac Contamination Since the Ban on its Veterinary Use

**Figure 9:**
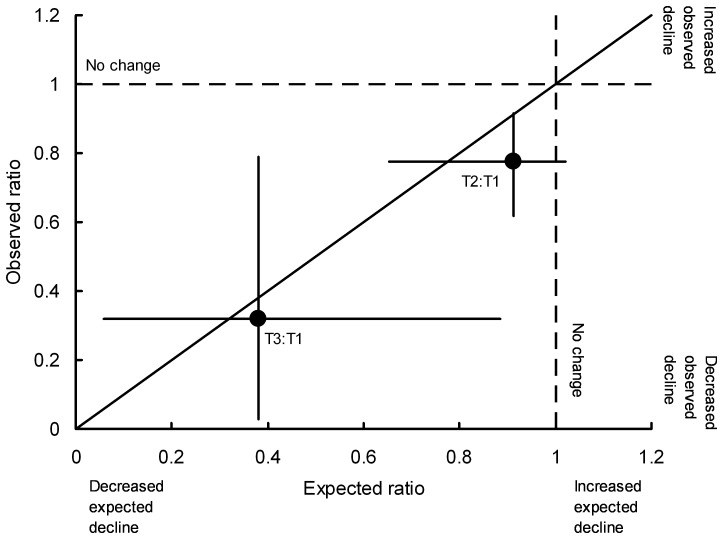
Change in the rate of decline of the *Gyps bengalensis* population in India observed and expected from the ban on diclofenac. The annual decline rate in two time periods after the ban on veterinary diclofenac, is shown relative to the decline rate before the ban. The horizontal axis shows the expected rate of decline calculated from the prevalence and concentration of diclofenac in liver samples from carcasses of domesticated ungulates sampled in April – December 2006 (T2) and in January 2007– December 2008 (T3), relative to the expected rate from samples collected before the ban (T1: May 2004– July 2005). The vertical axis shows the ratio of the vulture population decline rates at the mean sample collection dates of surveys T2 and T3 to that at T1, estimated from the cubic regression model fitted to the road transect data. The vertical and horizontal lines associated with each point show 95% bootstrap confidence limits. The diagonal line shows the result if the observed and expected ratios of decline rates were equal.

The rate of decline of *G. bengalensis* estimated from road transect surveys at the date of the survey of diclofenac in ungulate carcasses conducted after the ban (T2– samples collected April – December 2006) was 78% of the decline rate at the time of the survey before the ban (T1– May 2004– July 2005). The decline rate at the time of the survey conducted longer after the ban (T3– January 2007– December 2008) was 32% of the decline rate before the ban. These observed changes in vulture population decline rate are broadly similar to those expected from the data on diclofenac prevalence and concentration in ungulate carcasses (78% cf. 91% for the observed and expected T2 decline rate as a percentage of the T1 rate and 32% cf. 38% for the observed and expected T3 decline rate as a percentage of the T1 rate). The 95% confidence limits for these relative decline rates overlap the observed = expected relationship (Figure 9).

## Discussion

Our results indicate that the rapid declines of vulture populations in India and Nepal are slowing down. Changes in population trend must arise because of changes in demographic rates [19]. Our simulation modelling to determine the magnitude and duration of changes in annual survival and breeding success required to account for the slowing of the rate of population decline estimated from road transects in India indicates that large and rapid increases in survival and breeding success have occurred between 2005 and 2010. Simulated demographic rates were required to be unusually low during the most rapid phase of the declines in 2000–2003 and the observed change in the rate of population decline implies rapid increases in these rates therafter. The short duration of the period in which demographic rates appear to have increased is striking similar in both *Gyps bengalensis* and *G. indicus* when the same assumptions about the baseline adult survival rate are made, though uncertainty about this rate makes the precise period of change impossible to determine. The implied changes in demographic rates are sufficiently large and rapid that the most positive population changes within the confidence limits of the road transect estimates are rendered implausible.

Veterinary use of diclofenac was banned in India, Pakistan and Nepal in 2006. If this had resulted in an immediate removal of diclofenac from all carrion available to vultures, then the decline in the vulture population would have been expected to cease immediately. However, two surveys in India of diclofenac concentrations in liver samples taken from domesticated ungulates since the ban was introduced show that diclofenac was still present in a substantial proportion of ungulate carcasses until at least 2008, although its prevalence and concentration had both declined substantially [18]. Hence, the change in population trend coincides with partially effective voluntary efforts to reduce veterinary use of diclofenac in the Indian subcontinent and the gradual but incomplete implementation of the bans on its veterinary use. Although the ban in India has not yet been completely effective, our analyses suggest that the slowing of the vulture decline in India has been of similar magnitude to that expected from the partial removal of diclofenac from the birds’ food supply, as measured by surveys of diclofenac prevalence and concentration in carcasses of domesticated ungulates [18]. Although we draw attention to this consistency, we do not claim that our observations on their own constitute a rigorous measurement of the relative importance of diclofenac poisoning as a cause of the vulture decline, compared with other possible causes. However, we note that two previous analyses have examined the importance of all other potential causes of the decline, in combination, relative to diclofenac. These tests involved two approaches: (1) estimating from post mortem data on dead wild vultures the proportion of the excess mortality required to account for the observed rates of decline which is attributable to factors other than diclofenac poisoning [3], and (2) estimating the expected rate of vulture decline using measurements of diclofenac in liver samples from dead ungulates and comparing it with the observed rate of decline [14]. Both of these tests, which are based upon independent datasets, explicitly estimated the proportion of the excess mortality, required to cause the observed decline, that could be attributed to causes other than diclofenac. In the first test, the best estimate of the proportion of excess vulture mortality in India accounted for by causes other than diclofenac was 13–22% for *Gyps bengalensis* and 9–29% for *G. indicus* [3]. The ranges of values given are for different assumptions about the annual survival rate of adults. In the second test, the best estimate of the proportion of excess mortality of *Gyps bengalensis* in India accounted for by causes other than diclofenac was that there was no contribution from other causes. After allowing for uncertainty in the estimate, support for the hypothesis that diclofenac was the only cause of the decline was 84–98%, depending upon model assumptions [14].

We do not suggest that the change in diclofenac contamination is the only possible change in factors that might affect vulture populations during the period when the diclofenac ban was introduced. However, we are not aware of any other remedial conservation actions that have been introduced on a sufficient scale, though we acknowledge that a stringent test would require reliable quantitative data on changes over time in other potential factors affecting the rate of population decline and ways to convert them, using models, into expected effects on population trend. We do not know of such data or of practicable ways to obtain them.

We also cannot completely exclude the possibility that the slowing of the rate of decline has occurred, not because diclofenac contamination has been reduced, but because the remaining vultures are confined, to an increasing extent, to areas where little diclofenac is used or where much of the birds’ food comprises uncontaminated carcasses of wild ungulates. However, our examination of recent changes in population trends in Indian National Parks, where the majority of vultures counted in India in 2011 (Figure 6) were seen, does not support this alternative hypothesis. It is notable that there were rapid population declines in the early 2000s even in the National Parks where some vultures still remain and where they have recently increased slightly in numbers. This is presumably because vultures range over long distances from their breeding and roosting sites whilst foraging and are therefore exposed to diclofenac by feeding on contaminated carcasses of domesticated ungulates well beyond the boundaries of the parks. Gilbert et al. [20] found that five adult male *G. bengalensis*, satellite tagged in Pakistan in 2003–2004, ranged up to 25–316 km from their breeding or roosting sites (mean 160 km), even though supplementary food was provided near these sites during part of the period. Hence, there is likely to be a risk of diclofenac exposure for vultures in National Parks, even though feeding from carcasses of uncontaminated wild ungulates in the parks may reduce it. The smaller change in vulture density between 2000 and 2007 in and near the protected areas where vultures remained in 2011 than the change over this period in the index of the population as a whole may be because these areas have a less contaminated food supply. We conclude that the partial effectiveness of the ban on veterinary use of diclofenac is the most likely main cause of the slowing of the vulture decline in India.

A cessation of the previously observed decline in *G. bengalensis* was also recorded in western Nepal between 2009 and 2011, where previous counts had declined continuously between 2002 and 2009. The total number of birds counted in Nepal was much smaller than for India and the change in population trend was not statistically significant, but the similarity in the change in trend between India and Nepal is striking nonetheless. Data are not available for Nepal on the prevalence and concentration of diclofenac in ungulate carcasses before and after veterinary use of the drug was banned, so we cannot assess directly whether reduced contamination of the vultures’ food supply has contributed to this. However, a project led by Bird Conservation Nepal undertook widespread vulture conservation advocacy and awareness activities for vultures, as well as a programme of work to ensure the removal of veterinary stocks of diclofenac from pharmacies. This work was initiated in several lowland districts in Nepal in 2006 and has since spread to a programme across all western districts during 2008–2010. In 2011 surveys of 294 pharmacies in the lowlands of Nepal indicated that the alternative drug meloxicam, which has been promoted by conservation organizations as a safe alternative to diclofenac [21], [22], was found in 97% of pharmacies, whereas diclofenac was found in just 0.6% of pharmacies. Hence, the timing of the possible change in vulture population trend in western Nepal is broadly coincident with increased implementation of the ban on veterinary use of diclofenac and replacement of the latter drug with the vulture safe alternative meloxicam.

Although our findings are more encouraging than those from previous surveys, they do not mean that the future persistence of wild vultures in India and Nepal is assured. Because vultures are now so rare, the confidence intervals for our estimates of population trend are wide. Hence, their populations may well still be in decline, though at a significantly slower rate than before. The most recent surveys of diclofenac prevalence in ungulate carcasses [18] and of the availability of the drug in veterinary pharmacies in India [23], both of which were completed in 2008, showed that, although the prevalence and concentration of diclofenac in carcasses had declined substantially, it remained in widespread veterinary use at that time. Seventy percent of the Indian pharmacies selling veterinary NSAIDs were offering meloxicam and 36% were offering diclofenac, so diclofenac was available in considerably more pharmacies in India after the ban than it was in Nepal (97% and 0.6% respectively, see above). Continuation of monitoring of vulture populations and diclofenac prevalence and efforts to complete the removal of diclofenac and other toxic NSAIDs from the vultures’ food supply are essential. Such activities, along with the future release of vultures bred in conservation breeding centres, are required to ensure the establishment and persistence of self-supporting populations of wild vultures in the Indian subcontinent.
